# Cutting edge of genetically modified pigs targeting complement activation for xenotransplantation

**DOI:** 10.3389/fimmu.2024.1383936

**Published:** 2024-04-04

**Authors:** Qin Sun, Si-Yuan Song, Jiabao Ma, Danni Li, Yiping Wang, Zhengteng Yang, Yi Wang

**Affiliations:** ^1^ Department of Endocrinology, Sichuan Academy of Medical Sciences and Sichuan Provincial People’s Hospital, School of Medicine, University of Electronic Science and Technology of China, Chengdu, China; ^2^ Department of Neuroscience, Baylor College of Medicine, Houston, TX, United States; ^3^ School of Pharmacy, Guangxi University of Chinese Medicine, Nanning, China; ^4^ Department of Pharmacy, Longquanyi District of Chengdu Maternity & Child Health Care Hospital, Chengdu, China; ^5^ Department of Critical Care Medicine, Sichuan Academy of Medical Sciences and Sichuan Provincial People’s Hospital, School of Medicine, University of Electronic Science and Technology of China, Chengdu, China; ^6^ Clinical Immunology Translational Medicine Key Laboratory of Sichuan Province, Center of Organ Transplantation, Sichuan Academy of Medical Science and Sichuan Provincial People’s Hospital, Chengdu, Sichuan, China

**Keywords:** xenotransplantation, complement systems, genetically modified pigs, C3a, C3b

## Abstract

In the quest to address the critical shortage of donor organs for transplantation, xenotransplantation stands out as a promising solution, offering a more abundant supply of donor organs. Yet, its widespread clinical adoption remains hindered by significant challenges, chief among them being immunological rejection. Central to this issue is the role of the complement system, an essential component of innate immunity that frequently triggers acute and chronic rejection through hyperacute immune responses. Such responses can rapidly lead to transplant embolism, compromising the function of the transplanted organ and ultimately causing graft failure. This review delves into three key areas of xenotransplantation research. It begins by examining the mechanisms through which xenotransplantation activates both the classical and alternative complement pathways. It then assesses the current landscape of xenotransplantation from donor pigs, with a particular emphasis on the innovative strides made in genetically engineering pigs to evade complement system activation. These modifications are critical in mitigating the discordance between pig endogenous retroviruses and human immune molecules. Additionally, the review discusses pharmacological interventions designed to support transplantation. By exploring the intricate relationship between the complement system and xenotransplantation, this retrospective analysis not only underscores the scientific and clinical importance of this field but also sheds light on the potential pathways to overcoming one of the major barriers to the success of xenografts. As such, the insights offered here hold significant promise for advancing xenotransplantation from a research concept to a viable clinical reality.

## 1 Introduction

As of 2019, China’s organ donor registration boasted close to 1.7 million volunteers, a testament to its advancements in the field of organ transplantation. In that same year, China ranked as the world’s second-largest provider of allogeneic transplants, showcasing over 10,000 kidney and 5,000 liver transplants at the 4th China-International Organ Donation Conference (1). A significant policy shift in 2015 marked the transition to voluntary organ donations from Chinese citizens as the exclusive legal source for transplants (2), which, despite its ethical merits, has led to an even greater deficit in available human organs for transplantation and hindered research due to the scarcity.

**Figure 1:**
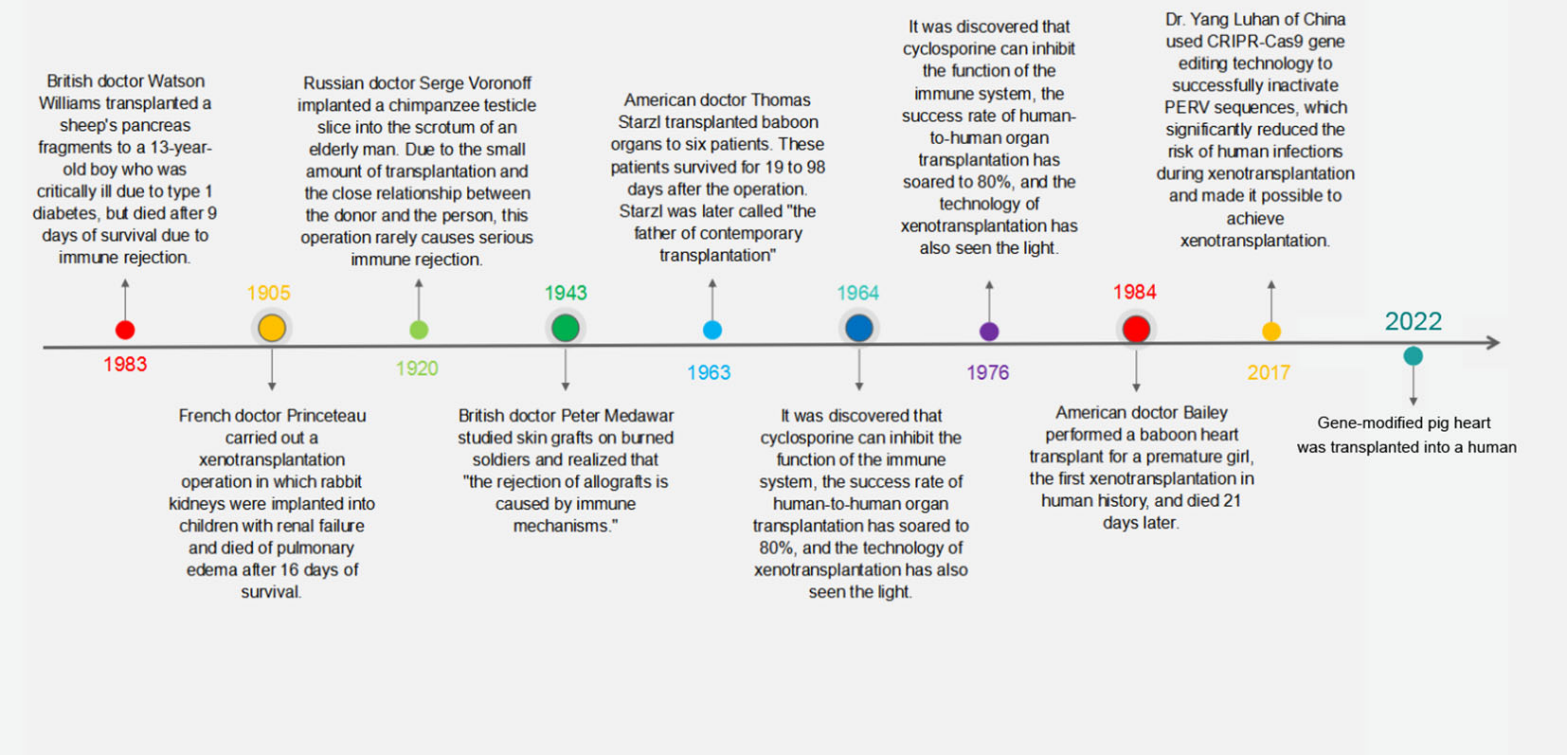
Milestones in the history of xenotransplantation. This timeline graphically represents the pivotal moments and groundbreaking achievements in the field of xenotransplantation from the early 20th century to modern day.

This backdrop has propelled xenotransplantation to the forefront as a promising solution to this shortage. Research in this domain has progressively moved toward identifying specific donor species, with primates being an initial choice due to their genetic closeness to humans. However, the use of baboon organs has consistently resulted in patient fatalities (3), steering the scientific focus toward pigs as suitable organ donors. Pigs, with their comparable organ size to humans and favorable breeding traits, are currently the focal point of xenotransplantation research (4–6). The journey of xenotransplantation, illustrated in **Figure 1**, is now directed toward the development of transgenic pigs, which are being heralded as the next step in transplantation science.

The hyperacute rejection of transplants, primarily driven by the complement system, has been a longstanding challenge. This system’s activation leads to the production of active compounds like C3a and C3b (7), which catalyze immune inflammation and graft endothelial thromboembolism. The discovery of α-Gal on graft surfaces as a trigger for complement activation has steered the development of α-Gal knockout (α-GalKO) pigs. Chinese research teams, such as the one led by Pandengke, have been at the helm of creating and refining α-Gal and β-Gal knockout pigs for several generations. A milestone was achieved in June 2020 with the cloning of a pig possessing triple knockouts, a significant leap made possible by gene editing technologies targeting the B4GalNT2 and CMAH genes (8).

The crux of this article revolves around the utilization of gene editing to modify pig donors, aiming to mitigate the issue of complement activation-induced hyperacute rejection post-xenotransplantation. We discuss dual approaches to this end: the genetic elimination of the α-Gal epitope from xenograft endothelium and the introduction of human complement regulatory proteins (hCRPs) into grafts via transgenesis. Additionally, we explore the pharmaceutical avenues developed to inhibit the complement system, a critical strategy to counter rejection in xenotransplantation.

## 2 Xenograft activates the complement system

Xenograft transplantation challenges the human immune system, particularly through the activation of the complement system, a sophisticated network of over 50 proteins crucial for the immune response (9). It can be activated via three primary pathways: the classical pathway (CL), the alternative pathway (AP), and the lectin pathway (MBL) (10), all leading to the potential destruction of the xenograft.

The classical pathway is initiated by the C1 complex binding to antigen-antibody complexes, leading to the activation of C4 and C2, and subsequently, the formation of C3 convertase (11). This enzyme is pivotal in cleaving C3 into C3a and C3b, with C3b joining with C4b2a to form C5 convertase, advancing the complement cascade (12). In contrast, the alternative pathway, triggered by substances like natural polysaccharides, relies on the spontaneous hydrolysis of C3 and the formation of a fluid-phase C3 convertase, leading to a modest production of C3b that enhances phagocytosis and anaphylatoxin production (13, 14). The lectin pathway starts with MBL binding to microorganism surface carbohydrates, recruiting MASP-1 and MASP-2 to form C3 convertase, mirroring the classical pathway’s initial steps (15, 16).

Xenotransplantation, especially from pig donors to primate recipients, introduces immunological hurdles due to the rapid complement-mediated response that often leads to hyperacute rejection (HAR), characterized by graft embolism and failure (7, 17). The presence of natural antibodies in the recipient binding to pig endothelial cell surface glycoproteins, such as α-galactosidase (α-Gal) and N-acetylneuraminic acid hydroxylase (Neu5Gc protein), activates the complement system, leading to clotting, vascular embolism, and graft failure (18, 19). Studies have shown that pig hearts transplanted into baboons are susceptible to this rapid rejection, with serum analysis revealing IgM-α-Gal antibodies bound to α-Gal, triggering the complement activation pathways (18, 19).

However, genetic engineering offers promising strategies to circumvent HAR by modifying donor pigs to reduce the human complement system’s activation effects on graft survival. Knocking out genes encoding heterologous endothelial antigens and creating transgenic pigs expressing hCRPs are at the forefront of these strategies (20). *In vitro* studies using pancreatic islets from α-GalKO pigs showed reduced antibody deposition and lower levels of complement activation, suggesting a diminished role of the lectin pathway in xenograft rejection (18, 19).

Further research into the immunological interactions between pig tissues and primate hosts has revealed that even in the absence of preformed natural antibodies, HAR can occur, potentially through the alternative complement pathway (21, 22). This indicates a complex interplay between the classical and alternative pathways in graft rejection, where the alternative pathway may exacerbate C3a deposition within grafts, amplifying inflammatory and immune responses (23).

Complement proteins C3a and C5a, along with the membrane attack complex formed via the classical and alternative pathways, play critical roles in xenograft tissue lysis. These proteins not only mediate inflammation but also activate coagulation cascades, contributing to the risk of thromboembolism in xenografts (24). Studies have shown that inflammation induced by complement activation can significantly reduce the expression of porcine thrombomodulin, an anti-inflammatory molecule, on vascular endothelial cells, highlighting the interconnectedness of inflammation and thrombosis in xenotransplantation (25).

Addressing the challenge of HAR in xenotransplantation requires innovative approaches to prevent complement activation. Genetic modifications in pig donors, such as eliminating α-Gal epitopes and introducing hCRPs, represent vital steps toward improving graft survival and reducing complement-mediated rejection risks. These strategies not only aim to mitigate the immediate immunological challenges but also open new avenues for long-term success in xenotransplantation, potentially transforming it into a viable solution for organ shortages (20).

## 3 Genetic modifications in pigs

**Figure 2:**
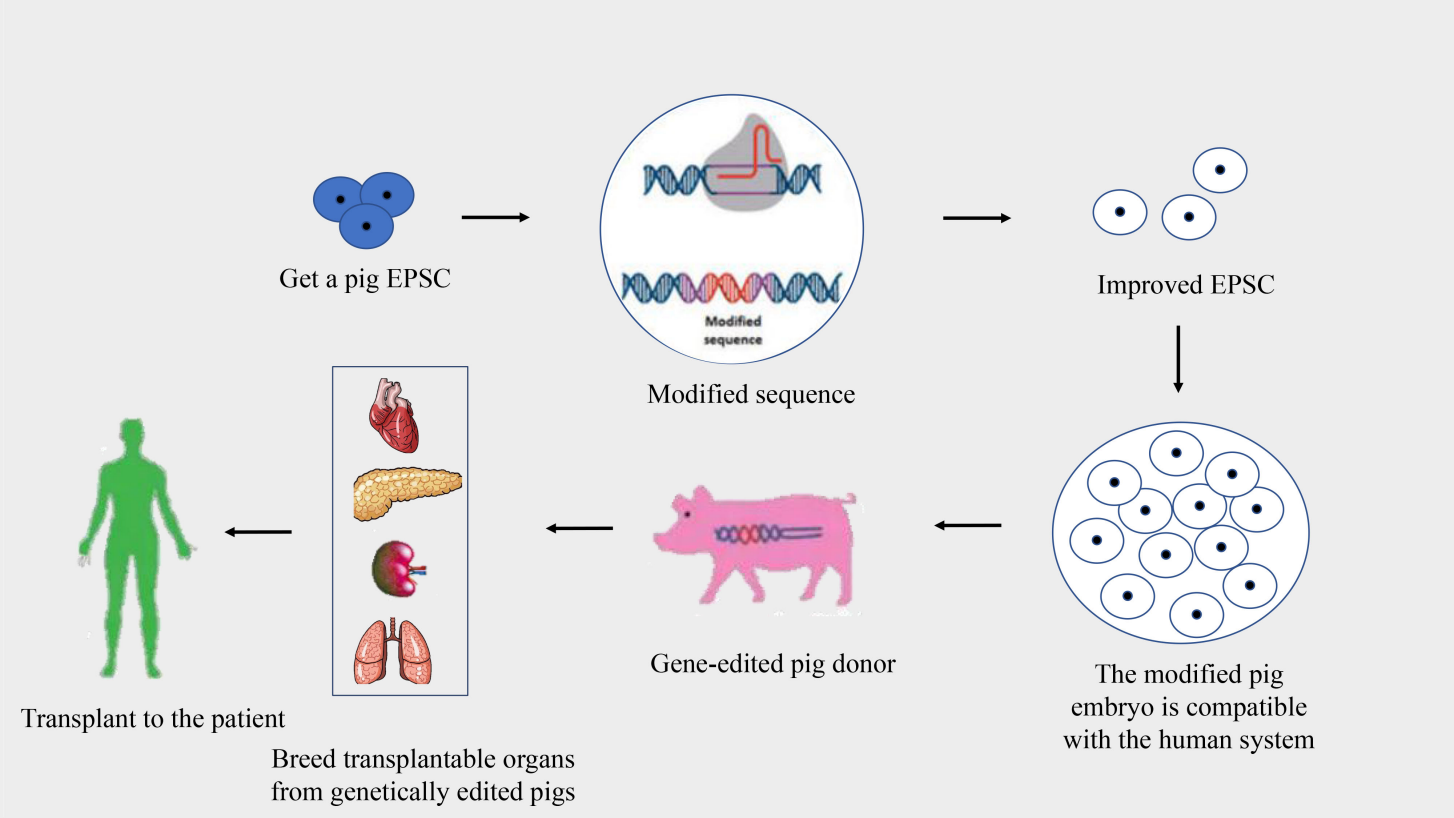
Process of creating gene-edited pig donors for xenotransplantation. This flowchart illustrates the stages of developing gene-edited pigs for organ donation to human recipients.

Pigs are optimal donors for xenotransplantation due to their genetic, physiological, and anatomical similarities to humans, alongside their capability for breeding in controlled environments (26, 27). Despite these advantages, the genetic differences between pigs and humans can lead to immunological discordance and potential organ rejection. Advancements in genetic engineering and somatic cell nuclear transfer have facilitated modifications to the pig genome to reduce organ immunogenicity, aiming to prevent the human immune system from rejecting pig organ transplants (27, 28) (**Figure 2**). This progress is pivotal in addressing immune rejections, with research exploring the growth of human organs within pigs through chimeric methods, although still predominantly in rodent models.

The risk of viral infection, particularly from porcine endogenous retroviruses (PERVs), represents a significant challenge in xenotransplantation (29). Strategies to mitigate this risk include breeding pigs in specific-pathogen-free (SPF) environments and selecting pigs free from PERV-C to reduce the risk of PERV-A/C-mediated transmission to humans (30). Although endogenous retroviruses remain inactive within their host species, causing no apparent disease, they could potentially become active and infectious upon transmission to a recipient (27, 31, 32). Immune molecular incompatibility poses another obstacle, with the immune system targeting foreign grafts, notably triggered by pre-existing natural xenoantibodies recognizing Gal epitopes (33–35). Genetically engineered pigs lacking alpha-1,3-Gal epitopes represent a crucial step toward overcoming HAR and other forms of immune rejection (27, 28).

Non-specific immune reactions, such as the instant blood-mediated inflammatory reaction (IBMIR), significantly challenge xenogeneic islet transplantation, leading to substantial graft loss (36). Addressing these reactions involves genetic modifications of donor animals, anticoagulation therapies, and the use of anti-inflammatory treatments to preserve graft integrity and prevent adaptive immune activation (37).

## 4 Genetic modification of pigs for xenotransplantation

The development of genetically engineered pigs marks a significant leap forward in addressing the challenges of xenotransplantation from pigs to primates. Through cutting-edge genome editing techniques, scientists have been able to introduce precise modifications into the pig genome to mitigate xenograft rejection and diminish the risk of interspecies infection (28). Among the most promising modifications are the disruption of the α-Gal and the incorporation of hCRPs, which have shown considerable promise in preclinical studies involving pig-to-non-human primate transplants.

Recent breakthroughs in gene editing, powered by artificial nuclease technologies, have significantly expanded the possibilities for generating gene-edited pigs. These technologies, including zinc finger nuclease (ZFN) (38), transcription activator-like effector nuclease (TALEN) (39), and the CRISPR/Cas system (40–43), have enabled not only simple gene knockouts and knock-ins but also complex multi-gene editing, precision point mutations, and conditional gene modifications. These advancements allow for gene editing at various developmental stages of pigs, offering new avenues for creating donor pigs with optimized genetic traits for xenotransplantation.

The hCRPs play a crucial role in maintaining the delicate balance between complement activation and inhibition. Proteins such as decay-accelerating factor (hDAF), membrane cofactor protein (hMCP), and reactive membrane cleavage inhibitor (hCD59) prevent unregulated complement activity, which could otherwise lead to continuous production of complement components and exacerbate endothelial damage in xenografts (44). The expression of these hCRPs in donor pigs can significantly reduce the risk of hyperacute rejection by limiting the formation of the membrane attack complex (MAC) and mitigating complement-mediated damage.

The application of DAF (CD55), a membrane component found on various human cells, has been explored for its potential to protect grafts from early rejection phases (45, 46). DAF can disrupt C3 convertases on the cell surface, effectively downregulating complement activation. Studies have demonstrated that expressing hDAF in pig islets and other tissues can enhance protection against human complement-mediated lysis and extend graft survival (47, 48). Similarly, the expression of human h-transferase, an inhibitor of the alternative complement pathway, has been shown to provide significant protection for xenografts against human complement attack, as evidenced by experiments with transgenic pig livers transplanted into baboons (49, 50). These genetic modifications underscore the potential of genetically engineered pigs to overcome some of the most significant barriers to successful xenotransplantation.

Membrane cofactor protein (MCP, CD46) plays a crucial role in preventing the amplification loop of C3b deposition mediated by alternative convertase. In an innovative approach, researchers employed α-GalKO pigs that were genetically modified to express human CD46 across all tissues, including the heart, exhibiting elevated levels of human CD46 expression. This genetic modification not only prevented B cell infiltration but also significantly reduced T cell activity in the peripheral blood of transplants, indicating an effective suppression of the T cell-mediated response to xenoantigens (51).

Human CD59 serves as a protective mechanism against autologous cell damage by the human complement system, specifically by inhibiting the formation of the membrane attack complex (MAC) during the final stage of complement activation (7, 52). Utilizing embryonic germ (EG) cells, which unlike somatic cells can proliferate indefinitely while remaining undifferentiated, Hosup Shim (53) developed a method to create transgenic pigs capable of expressing human CD59. These EG cells, derived from primordial germ cells (PGC) (54), were genetically modified with a 456 bp fragment of the hCD59 gene, encompassing the entire coding region, obtained from human fibroblast genes (55). Post-transfection into porcine EG cells (56), these modified cells exhibited significantly higher mitochondrial activity when exposed to human serum containing complement, compared to non-transgenic controls, demonstrating enhanced survival under HAR conditions.

The development of multi-transgenic pigs offers a promising strategy to mitigate xenograft damage more effectively. For instance, pig cells expressing human CD59 have shown increased resistance to lysis by human macrophages (57). Furthermore, the expression of α1,2-fucosyltransferase (H-transferase, HT), alongside the knockout of the α1,3-galactosyltransferase (GT) gene, presents a viable alternative strategy. Combining gene edits to express both hCD59 and human HT, or to achieve α-GalKO, enhances the protective effects against human serum, thereby improving cell and organ survival post-transplantation (58). Transgenic pigs expressing human CD55, CD59, and H-Transferase have shown significant reduction in complement-mediated graft destruction (50), although these modifications alone could not completely prevent humoral rejection, characterized by antibody deposition and thrombotic microangiopathy. This suggests that while significant strides have been made, further research is necessary to minimize rejection mechanisms in xenotransplantation (28).

## 5 Complement system target drugs for transplantation therapy

The complement system plays a crucial role in innate immunity and immune regulation, protecting against infections and participating in various physiological and pathological processes (59). Despite its protective functions, dysregulated complement activation can contribute to detrimental effects, including inflammation and tissue damage. A deeper understanding of the complement system’s components and mechanisms has spurred the development of therapeutic drugs aimed at modulating complement activity. These drugs target various complement pathways, offering potential treatments for infectious, inflammatory, traumatic, cancerous, autoimmune, or age-related conditions, as well as preventing transplant rejection (60).

Eculizumab, the first drug targeting the complement system, has revolutionized the treatment landscape for diseases like paroxysmal nocturnal hemoglobinuria (PNH), significantly improving patient outcomes (59, 61). In the context of organ transplantation, the complement system is implicated in several complications, including ischemia-reperfusion injury and antibody-mediated rejection. Therapeutics such as C1-1NH (Cinryze, Berinert, Ruconest, Cetor) and Soliris are making their way into clinical practice, showing promise but with varying efficacy levels (62). Future research is needed to identify the most effective complement inhibitors and devise optimal treatment strategies. The development programs for inhibitors targeting over a dozen distinct complement pathways are summarized, with some already undergoing clinical trials in both healthy volunteers and patients (62–64). This broad spectrum of complement-targeted therapies underscores the system’s significance across a range of medical conditions and its potential as a therapeutic target in transplant medicine, where controlling complement activation could mitigate transplant rejection and improve graft survival.

## 6 Conclusions and perspective

The critical shortage of human organs for transplantation is a global challenge, and xenotransplantation has emerged as a promising approach to address this dilemma. Genetically engineered pigs are at the forefront of donor options in xenotransplantation, offering a viable solution to the organ shortage crisis. Advances in gene editing technologies, such as CRISPR/Cas9, TALEN, and somatic cell nuclear transfer (SCNT), have significantly propelled xenotransplantation research forward, enabling precise genetic modifications in pig donors.

The complement system plays a dual role in xenotransplantation: it is a key player in the immune response against porcine endothelial cells following the binding of anti-porcine antibodies and contributes to ischemia-reperfusion injury (IRI). Additionally, its involvement in coagulation, inflammation, and the adaptive immune response adds layers of complexity to its function in xenograft rejection. Despite these immunobiological challenges, the advent of genetically modified pigs, alongside an expanding array of immunosuppressants and anti-inflammatory medications, is progressively overcoming the hurdles faced by xenotransplantation.

Current genetic engineering efforts targeting complement regulatory mechanisms have effectively mitigated concerns related to complement activation. However, there remains a potential necessity for anti-complement and anti-inflammatory interventions, especially in acute settings, to ensure the long-term success and acceptance of xenotransplantation as a feasible solution to the organ shortage crisis.

## Author contributions

QS: Writing - original draft. QY: Writing – original draft. S-YS: Writing – original draft. JM: Writing – original draft. DL: Writing – original draft. YPW: Writing – original draft. ZY: Funding acquisition, Writing – review & editing. YW: Funding acquisition, Writing – review & editing.

## References

[1] National Bureau of Statistics of China. National Economy was Generally Stable in 2019 with Main Projected Targets for Development Achieved, (Beijing, China: National Bureau of Statistics of China) (2020). Available at: https://www.stats.gov.cn/english/PressRelease/202001/t20200117_1723398.html.

[2] Li J-H Xu X Wang Y-F Xie H-Y Chen J-Y Dong N-G. Chinese expert consensus on organ protection of transplantation (2022 edition). Hepatobiliary & Pancreatic Diseases International. (2022) 21(6):516–526. doi: 10.1016/j.hbpd.2022.10.010 36376226

[3] Cooper DKC. A brief history of cross-species organ transplantation. Proc (Bayl Univ Med Cent). (2012) 25(1):49–57. doi: 10.1080/08998280.2012.11928783 PMC3246856 22275786

[4] Reardon S. First pig-to-human heart transplant: what can scientists learn? Nature. (2022) 601:305–6. doi: 10.1038/d41586-022-00111-9 35031782

[5] Montgomery RA Stern JM Lonze BE Tatapudi VS Mangiola M Wu M. Results of two cases of pig-to-human kidney xenotransplantation. N Engl J Med. (2022) 386:1889–98. doi: 10.1056/NEJMoa2120238 35584156

[6] Porrett PM Orandi BJ Kumar V Houp J Anderson D Cozette Killian A. First clinical-grade porcine kidney xenotransplant using a human decedent model. Am J Transplant. (2022) 22:1037–53. doi: 10.1111/ajt.16930 35049121

[7] Tan LA Yu B Sim FC Kishore U Sim RB. Complement activation by phospholipids: the interplay of factor H and C1q. Protein Cell. (2010) 001:1033–49. doi: 10.1007/s13238-010-0125-8 PMC4875149 21153520

[8] Zhang Y Pan D Sun X Sun G Wang X Liu X. Production of porcine cloned transgenic embryos expressing green fluorescent protein by somatic cell nuclear transfer. Sci China Ser C. (2006) 49):1–8. doi: 10.1007/s11427-005-0071-5 16704120

[9] Liu C-CM Susan M Kao AH Navratil JS Ahearn JM. Cell-bound complement biomarkers for systemic lupus erythematosus: from benchtop to bedside. Rheum Dis Clin North Am. (2010) 36:161–72. doi: 10.1016/j.rdc.2009.12.003 PMC2837510 20202597

[10] Roos A Daha M. Antibody-mediated activation of the classical complement pathway in xenograft rejection. Transplant Immunol. (2002) 9:257–70. doi: 10.1016/S0966-3274(02)00042-4 12180840

[11] Wallis R Mitchell DA Schmid R Schwaeble WJ Keeble AH. Paths reunited: initiation of the classical and lectin pathways of complement activation. Immunobiology. (2010) 1:1–11. doi: 10.1016/j.imbio.2009.08.006 PMC2824237 19783065

[12] Ballow M. C1-Bypass complement-activation pathway in patiente with chronic urticaria and angiosoelig. Lancet. (1975) 306:248–50. doi: 10.1016/S0140-6736(75)90963-0 49798

[13] Gtze O Müller-Eberhard HJ. The alternative pathway of complement activation. Adv Immunol. (1976) 24:1–35. doi: 10.1016/s0065-2776(08)60328-4 798473

[14] Merle NS Elizabeth CS Veronique FB Roumenina LT. Complement system part I a. Front Immunol. (2015) 6:1–30. doi: 10.3389/fimmu.2015.00257 25657648 PMC4302982

[15] Cooper DKC Sachs DH Colvin RB Shimizu A Hisashi Y Yamada K. Rejection of cardiac xenografts transplanted from αr,3-3omsplantedFDSLHSCRHJ genesplantedF (GalT-alT pigs to baboons. Am J Transplant. (2008) 8:2516–26. doi: 10.1111/j.1600-6143.2008.02444.x PMC2836186 19032222

[16] Kaplon RJ Platt JL Kwiatkowski PA Edwards NM Xu HE Shah AS. Absence of hyperacute rejection in pig-to-primate orthotopic pulmonary xenografts. Transplantation. (1995) 59:410. doi: 10.1097/00007890-199502150-00017 7871572

[17] Chen RH Kadner A Mitchell RN Adams DH. Mechanism of delayed rejection in transgenic pig-to-primate cardiac xenotransplantation. J Surg Res. (2000) 90:119–25. doi: 10.1006/jsre.2000.5864 10792951

[18] Thompson P Badell I Lowe M Cano J Song M Leopardi F. Islet xenotransplantation using gal-deficient neonatal donors improves engraftment and function. other. (2011) 11:1–20. doi: 10.1111/j.1600-6143.2011.03720.x PMC3226931 21883917

[19] Broom C Uknis ME. Methods of treating antibody-mediated rejection in organ transplant patients with C1-esterase inhibitor. (Australia: Patent) (2018).

[20] Butler JR Ladowski JM Martens GR Tector M Tector AJ. Recent advances in genome editing and creation of genetically modified pigs. Int J Surgery. (2015) 23:217–22. doi: 10.1016/j.ijsu.2015.07.684 26231992

[21] Suckfüll M Müdsam M Pieske O Enders G Babic R Hammer C. Immunohistological studies of complement activation after xenogeneic perfusion of a working heart model. Transplant Int. (1994) 7:324–8. doi: 10.1111/j.1432-2277.1994.tb01241.x 7993568

[22] Forty J Hasan R Cary N White DJ Wallwork J. Hyperacute rejection of rabbit hearts by human blood is mediated by the alternative pathway of complement. Transplant Proc. (1992) 24:488. 1566398

[23] Platts-Mills TAE Ishizaka K. Activation of the alternative pathway of human complement by rabbit cells. J Immunol. (1974) 113:348–58. doi: 10.4049/jimmunol.113.1.348 4134064

[24] Li Y Gong P Kong C Tian X. Bufalin engages in RIP1-dependent and ROS-dependent programmed necroptosis in breast cancer cells by targeting the RIP1/RIP3/PGAM5 pathway. Anti Cancer Drugs. (2019) 30:e0770. doi: 10.1097/CAD.0000000000000770 30829654

[25] Ochando J Ordikhani F Boros P Jordan S. The innate immune response to allotransplants: mechanisms and therapeutic potentials. Cell Mol Immunol. (2019) 16):350–6. doi: 10.1038/s41423-019-0216-2 PMC6462017 30804476

[26] Heng Z Kaixiang X Ninglin F Hongye Z Hongjiang W. Construction and current status of gene-edited xenotransplantation pigs. Electronic J Pract Organ Transplantation. (2018) 6:412–8.

[27] Hryhorowicz M Zeyland J Słomski R Lipiński D. Genetically modified pigs as organ donors for xenotransplantation. Mol Biotechnol. (2017) 59(9-10):435–44. doi: 10.1007/s12033-017-0024-9 PMC5617878 28698981

[28] Klymiuk N Aigner B Brem G Wolf E. Genetic modification of pigs as organ donors for xenotransplantation. Mol Reprod Dev. (2010) 77:209–21. doi: 10.1002/mrd.21127 19998476

[29] Specke V Rubant S Denner J. Productive infection of human primary cells and cell lines with porcine endogenous retroviruses. Virology. (2001) 285:177–80. doi: 10.1006/viro.2001.0934 11437652

[30] Patience C Switzer WM Takeuchi Y Griffiths DJ Weiss RA. Multiple groups of novel retroviral genomes in pigs and related species. J Virol. (2001) 75:2771–5. doi: 10.1128/JVI.75.6.2771-2775.2001 PMC115901 11222700

[31] Denner J. Porcine Endogenous Retroviruses and Xenotransplantation. Viruses. (2021) 13(11):2156. doi: 10.3390/v13112156 34834962 PMC8625113

[32] Denner J. Recombinant porcine endogenous retroviruses (PERVviruse a new risk for xenotransplantation? Xenotransplantation. (2010) 17:120–0. doi: 10.1111/j.1399-3089.2010.00573_21.x 18584115

[33] Ibrahim Z Busch J Awwad M Wagner R Wells K Cooper DKC. Selected physiologic compatibilities and incompatibilities between human and porcine organ systems. Xenotransplantation. (2010) 13:488–99. doi: 10.1111/j.1399-3089.2006.00346.x 17059572

[34] Guoling L Zhiqian X Huaqiang Y Zhenfang W. Research progress of transgenic and gene-edited pigs. J South China Agric Univ. (2019) 40(5):91–101.

[35] Tanihara F Hirata M Otoi T. Current status of the application of gene editing in pigs. J Reprod Dev. (2021) 67(3):177–87. doi: 10.1262/jrd.2021-025 PMC8238678 33840678

[36] Matsumoto S Tomiya M Sawamoto O. Current status and future of clinical islet xenotransplantation. J Diabetes. (2016) 8(4):483–93. doi: 10.1111/1753-0407.12395 26987992

[37] Zhengzhao L Tian H Zhiming C Lisha M. Research progress of porcine islet xenotransplantation. Organ transplant. (2017) 008:246–50.

[38] Miller JC Holmes MC Wang J Guschin DY Lee YL Rupniewski I. An improved zinc-finger nuclease architecture for highly specific genome editing. Nat Biotechnol. (2007) 25:778–85. doi: 10.1038/nbt1319 17603475

[39] Schmid-Burgk JL Schmidt T Kaiser V Höning K Hornung V. A ligation-independent cloning technique for high-throughput assembly of transcription activator-like effector genes. Nat Biotechnol. (2013) 31(1):76–81. doi: 10.1038/nbt.2460 PMC4142318 23242165

[40] Mali P Yang L Esvelt KM Aach J Guell M Dicarlo JE. RNA-guided human genome engineering *via* cas9. Science. (2013) 339:823. doi: 10.1126/science.1232033 23287722 PMC3712628

[41] Hsu PD Lander ES Zhang F. Development and applications of CRISPR-cas9 for genome engineering. (2014) 157(6):1262–78. doi: 10.1016/j.cell.2014.05.010 PMC4343198 24906146

[42] Gaj T Gersbach CA Barbas CF. ZFN, TALEN, and CRISPR/Cas-based methods for genome engineering. Trends Biotechnol. (2013) 31(7):397–405. doi: 10.1016/j.tibtech.2013.04.004 PMC3694601 23664777

[43] Yaoqiang H Guoling L Huaqiang Y Zhenfang W. Application of gene-edited pigs in biomedical research. Genetic. (2018) v.40:30–44.

[44] Roumenina LT Zuber J Frémeaux-Bacchi V. Physiological and therapeutic complement regulators in kidney transplantation. Curr Opin Organ Transplant. (2013) 18:421–9. doi: 10.1097/MOT.0b013e32836370ce 23838647

[45] Kinoshita T. Distribution of decay-accelerating factor in the peripheral blood of normal individuals and patients with paroxysmal nocturnal hemoglobinuria. J Exp Med. (1985) 162:75–92. doi: 10.1084/jem.162.1.75 2409211 PMC2187705

[46] Asch AS. Decay-accelerating factor is present on cultured human umbilical vein endothelial cells. J Exp Med. (1986) 163:221–6. doi: 10.1084/jem.163.1.221 PMC2188008 2416869

[47] Yamamoto T Iwase H King TW Hara H Cooper DKC. Skin xenotransplantation: Historical review and clinical potential. Burns. (2018) 44(7):1738–1749. doi: 10.1016/j.burns.2018.02.029 PMC6160369 29602717

[48] Schmidt P Goto M Mauff BL Anegon I Korsgren O. Adenovirus-mediated expression of human CD55 or CD59 protects adult porcine islets from complement-mediated cell lysis by human serum. Transplantation. (2003) 75:697–702. doi: 10.1097/01.TP.0000053249.39753.D6 12640312

[49] Young-Hee J Chi-Hun P Gun-Hyuk J Yeun-Ik J In-Sung H Yeon-Woo J. Production of multiple transgenic yucatan miniature pigs expressing human complement regulatory factors, human CD55, CD59, and H-transferase genes. PloS One. (2013) 8:e63241. doi: 10.1371/journal.pone.0063241 23704897 PMC3660325

[50] Ramírez P Montoya MJ Ríos A Palenciano CG Majado M Chávez R. Prevention of hyperacute rejection in a model of orthotopic liver xenotransplantation from pig to baboon using polytransgenic pig livers (CD55, CD59, and H-transferase). Transplant Proc. (2005) 37:4103–6. doi: 10.1016/j.transproceed.2005.09.186 16386637

[51] Mohiuddin MM Corcoran PC Singh AK Azimzadeh A Hoyt RF Jr. Thomas ML. B-cell depletion extends the survival of GTKO.hCD46Tgpig heart xenografts in baboons for up to 8 months. Am J Transplantation. (2012) 12:763–71. doi: 10.1111/j.1600-6143.2011.03846.x PMC4182960 22070772

[52] Kimberley FC Sivasankar B Morgan BP. Alternative roles for CD59. Mol Immunol. (2007) 44:73–81. doi: 10.1016/j.molimm.2006.06.019 16884774

[53] Ahn KS Ji YW Park JK Sorrell AM Heo SY Kang JH. Production of human CD59-transgenic pigs by embryonic germ cell nuclear transfer. Biochem Biophys Res Commun. (2010) 400:667–72. doi: 10.1016/j.bbrc.2010.08.125 20816662

[54] Uszewski KM. Control of the complement system. Adv Immunol. (1996) 61:201–83. doi: 10.1016/S0065-2776(08)60868-8 8834497

[55] Ji YW Ahn KS Sorrell AM Shin S Shim H. Cytolytic assessment of hyperacute rejection and production of nuclear transfer embryos using hCD46-transgenic porcine embryonic germ cells. Zygote. (2009) 17:101–8. doi: 10.1017/S096719940800511X 19063773

[56] Lee JH Lee HJ Nahm KM Jeon HY Hwang WS Paik NW. Effects of combined expression of human complement regulatory proteins and H-transferase on the inhibition of complement-mediated cytolysis in porcine embryonic fibroblasts. Transplant Proc. (2006) 38:1618–21. doi: 10.1016/j.transproceed.2006.02.129 16797369

[57] Shim H. Isolation of pluripotent stem cells from cultured porcine primordial germ cells. Biol Reproduction. (1997) 47:1089–95. doi: 10.1095/biolreprod57.5.1089 9369175

[58] Chen CG Salvaris EJ Romanella M Aminian A Pearse MJ. Transgenic expression of human alpha1,2-fucosyltransferase (H-transferase) prolongs mouse heart survival in an ex vivo model of xenograft rejection. Transplantation. (1998) 65:832. doi: 10.1097/00007890-199803270-00011 9539096

[59] Rother RP Rollins SA Mojcik CF Brodsky RA Bell L. Discovery and development of the complement inhibitor eculizumab for the treatment of paroxysmal nocturnal hemoglobinuria. Nat Biotechnol. (2007) 25:1256. doi: 10.1038/nbt1344 17989688

[60] Melis JPM Strumane K Ruuls SR Beurskens FJ Schuurman J Parren PWHI. Complement in therapy and disease: Regulating the complement system with antibody-based therapeutics. Mol Immunol. (2015) 67:117–30. doi: 10.1016/j.molimm.2015.01.028 25697848

[61] Woodruff TM Nandakumar KS Tedesco F. Inhibiting the C5-C5a receptor axis. Mol Immunol. (2011) 48:1631–42. doi: 10.1016/j.molimm.2011.04.014 21549429

[62] Ricklin D Lambris JD. New milestones ahead in complement-targeted therapy. Semin Immunol. (2016) 28(3):208–22. doi: 10.1016/j.smim.2016.06.001 PMC5404743 27321574

[63] Morgan BP Harris CL. Complement, a target for therapy in inflammatory and degenerative diseases. Nat Rev Drug Discovery. (2015) 14(12):857–77. doi: 10.1038/nrd4657 PMC7098197 26493766

[64] Risitano AM Marotta S. Therapeutic complement inhibition in complement-mediated hemolytic anemias: Past, present and future. Semin Immunol. (2016) 28(3):223–40. doi: 10.1016/j.smim.2016.05.001 27346521

